# Loss of a Parent in Disney and Pixar Films: What We Can Learn?

**DOI:** 10.1177/00302228241295344

**Published:** 2024-10-28

**Authors:** Corina Sgier, Mirriam Tyebally Fang, Andrea Glässel, Settimio Monteverde

**Affiliations:** 1 Institute of Biomedical Ethics and History of Medicine (IBME), 156378 University of Zurich, Zurich, Switzerland; 2 Institute of Public Health (IPH), Department of Health Sciences, Zurich University of Applied Sciences (ZHAW), Winterthur, Switzerland; 3 School of Health Professions, Bern University of Applied Science, Bern, Switzerland

**Keywords:** parental death, parentally bereaved children, disney and pixar film, QUAGOL, narration

## Abstract

Early parental loss is a tragic experience for children causing complex reactions to the loss. Providing a supportive environment where they can express their feelings is crucial to help them cope with this challenging experience. This study analyses the depiction of parental death in animated films by Disney and Pixar using a multimethod design and including the QUAGOL approach. We identified 13 films showing the death of one or both parents. The qualitative analysis of the films, published from 1937 until 2022, revealed seven concepts that potentially affect the way children see their grieving process reflected in the films: The representational techniques, finding protection and relationships, searching for identity, being different and having alternative skills, talking about death, dealing with emotions and coping with the loss. The identified films can be used to open a conversation with children who have lost one or both parents to discuss their situation.

## Introduction

Losing a loved one by death is a profound life-altering event (Champion & Kilcullen, 2023; Dutta et al., 2019). The death of a parent is considered one of the most stressful and traumatic experiences for children and adolescents (Alvis et al., 2023; Haine et al., 2008; Luecken & Roubinov, 2012; Yeaworth et al., 1980). The bonds we share with loved ones play a significant role in shaping our identity, enriching a sense of belonging, and filling our lives with meaning and purpose (Champion & Kilcullen, 2023; Shear, 2015). The loss of a loved one can lead us to question our existence and the meaning behind it. However, children’s perception of the death of parents can vary widely depending on their age (Høeg et al., 2017; Kennedy et al., 2018; Menendez et al., 2020; Stroebe et al., 2007), social and cultural inclusion (Menendez et al., 2020; Stroebe et al., 2007), socio-economic factors such as poverty (Stroebe et al., 2007; Wimpenny et al., 2007), and circumstances, such as received family support received surrounding the loss (Høeg et al., 2017; Stroebe et al., 2007; Wimpenny et al., 2007). Similarly, children’s reactions to the death of a parent can vary in complexity. Providing a supportive environment where they can express their feelings is crucial to help them navigate this challenging experience (Colman et al., 2014). At the age of ten, most children have fully grasped that death is inevitable, permanent, and irreversible. However, many children younger than ten years might only possess a limited comprehension of death due to their cognitive immaturity (Colman et al., 2014).

One in five children who have lost a parent develops a psychiatric disorder, such as anxiety, regression, or depression (Dowdney, 2000). The process of constructing the events into a meaningful narrative enables the child to transform this painful experience into a meaningful memory (Cohen & Mannarino, 2015). Furthermore, the process of integrating such an experience into one’s life story has been shown to strengthen resilience (Christ & Christ, 2006; Hewson et al., 2023; Rauch et al., 2002; Ryan & Ripley, 2023).

Conversations about death and dying, especially with children, tend to be limited in society due taboos surrounding the topic (Fearnley, 2010; Wray et al., 2022). Children who have lost one or both parents, by contrast, often want to talk clearly and directly about their loss. Adult caregivers, whether professionally or personally connected to the child, play an important role in supporting a child’s psychological well-being (Joy et al., 2024; Martinčeková et al., 2020; Wood et al., 2006), but they can miss this opportunity by trying to avoid feelings of discomfort and feeling ill-equipped to have these conversations (Fearnley, 2010). This reticence then contributes to other factors limiting the child’s ability to engage in the process of constructing a meaningful narrative out of their loss. Conversely, more frequent portrayal of the topic in the media, whether factual or fictional, could provide children with numerous opportunities to reflect on death and dying (Weiss, 2006), process the death they experience, and subsequently explore healthy ways of coping (Christ & Christ, 2006; Fearnley, 2010; Rauch et al., 2002), among them conversations with caretaking adults. This study explores the potential of popular films, specifically those made by Disney and Pixar, to support this process.

The use of various media, to explore behavioural models of coping with death and dying is supported by “social learning theory” (Bandura & Walters, 1977). Social learning theory states that we learn best through observation of others, which requires attention, memory, repetition, and motivation. This can be usefully supported using visual media. Social learning theory is embedded in the concept of “reciprocal determinism”, which states that humans influence the environment as much as the environment influences them (Bandura & Walters, 1977).

Using popular children’s films such as those from Disney and Pixar can be a creative way to open a conversation with children who have lost one or both parents. These films often address themes of loss, importance of the family, and resilience, which may provide a relatable starting point as a bridge to discussing their situation. Children can explore their feelings about death and dying by identifying with the characters in these films (Alvis et al., 2023; DiBartolo & Seldomridge, 2009; Graham et al., 2018; Jaén Portillo, 2024). With this in mind, this paper explores how popular children’s Disney and Pixar films can be used to gain access to the children’s emotional world and open a discussion and of the painful process of grieving the loss of one or both parents. Its specific aims are to:

a. Identify and describe how the death of a parent is portrayed in each film.
b. Describe how the child protagonist is affected by the loss at the moment of death and in future life.
c. Describe the role of the bereaved protagonist’s social environment on their ability to cope with this difficult situation.
d. Analyse the portrayal of the above for their potential benefit to grieving families.

## Methods

### Study Design

The study was conducted using a multimethod research design with two parts. The first part was a quantitative review of original Disney and Pixar films in the English language to identify films depicting parental death that fulfilled our inclusion criteria. The second part was a qualitative, interpretative-constructivist analysis, in which six researchers analysed the identified films using the Qualitative Analysis Guide of Leuven (QUAGOL) (Dierckx de Casterlé et al., 2012).

### Quantitative Review of Subject-Related Films

#### Search Strategy and Eligibility Criteria

Tenzek and Nickels (2019), which analyses Disney and Pixar animated films up to 2015 for the depiction of an end-of-life scene, provided the foundation for this study. It included all of the films identified in Tenzek & Nickels, while adding to these later releases up to and including 2022. As in Tenzek & Nickels, only films that were produced exclusively by Disney and Pixar were considered. The list of all films screened (*n* = 75) can be found in Appendix 1. The inclusion criteria were the identifiable loss by death of at least one parent sustained by a child, adolescent, or young adult (maximum age 24 years) during the film. The age of the bereaved character could be derived implicitly or shown explicitly in the film. The age limit of 24 was chosen in accordance with the MeSH (National Library of Medicine, 2009) definition of young adults as persons between 19 and 24 years old. This age limit also corresponds to the maximum age up to which children in Switzerland, where the study was conducted, receive financial support from the state if they suffer the loss of a parent. The state thus recognises and justifies their extended need for support. The exclusion criteria were films that featured the death of a significant caregiver or guardian who was not a parent, and those in which the orphan only casually recalled that a parent had died earlier in the orphan’s life.

#### Film Selection and Data Extraction

The lead researcher screened all films that met the inclusion criteria. In the cases of films in which inclusion or exclusion was uncertain, the research team was consulted and a consensus was reached. Data extraction occurred in two steps. In the first step, descriptive data were collected by the lead researcher, including the year of release, title of the film, length of the film, age rating, and the descriptions (see Table 1). In the second step, two researchers individually presented a descriptive overview of how death is portrayed in the respective films and presented this using deductive content analysis according to the five categories of death in Disney films, as proposed by Cox et al. (2005) and listed in Table 2.

**Table 1.** Overview of Included films.

Year Released	Title of the movie	Length of films, minutes	Age rating	Film description
1942	Bambi	68	0	**Bambi**, a young deer, discovers the mysterious wonders of nature with the clever rabbit Thumper and the shy skunk Flower, and the three become inseparable friends.
1981	The Fox and the Hound	83	0	**Tod,** a red fox and Copper, a hunting dog, cultivate a close and intense friendship. They romp through the forest together and have no idea that one day they will face each other as enemies.
1994	The Lion King	88	0	The small, courageous lion cub **Simba** seeks his place in the “circle of life”. He grows up under the good-natured care of his father Mufasa to later take his place as king. On the long journey through many challenges to reach this goal, he is helped by his funny friends Timon, a meerkat, and Pumbaa, a warthog.
1996	The Hunchback of Notre Dame	91	6	**Quasimodo**, a physically deformed young bell-ringer in his bell tower, longs to take part in the Feast of Fools. When he dares to do so, he finds acceptance and friendship with the beautiful and spirited Esmeralda. When she gets into trouble, Quasimodo wants to protect her and the two become involved in an exciting adventure.
1998	Mulan	88	0	The brave girl Mulan goes to war disguised as a man to replace her sick father. The cheeky dragon Mushu and the lucky cricket Cri-Kee stand by her side in the training camp. Mulan meets **Captain Li Shang** and learns to make her own way.
1999	Tarzan	88	0	**Tarzan** is raised as a human boy in a loving gorilla family. With his friends, the cheeky monkey Terk and the over-anxious elephant Tantor, things get rough before Tarzan can become master of the jungle.
2001	Atlantis: The Lost Empire	96	6	Young linguist **Milo** Thatch embarks on a dangerous search for the long-lost Atlantis. Milo’s expectations are exceeded when he and the fascinating Atlantean princess **Kida** explore the breathtaking underwater world and try to save it from destruction.
2003	Finding Nemo	96	0	The cheeky clownfish boy **Nemo** gets captured for an aquarium, and his anxious father Marlin has many adventures searching for him. On his journey to Sydney, where Nemo is stuck in a dentist’s aquarium, he meets the forgetful Dory and pseudo-vegetarian sharks.
2003	Brother Bear	85	0	The Indian boy **Kenai** is turned into a bear and accompanies the little bear **Koda** on his way to the mysterious mountain where he can become human again. The two are accompanied by two quirky moose brothers and Kenai soon sees the world through completely different eyes.
2009	The Princess and the Frog	97	0	**Tiana,** a pretty waitress, lives in New Orleans. She wants nothing more than to have her own restaurant. When she kisses a frog, she also turns into a frog. An adventurous search for the voodoo priestess begins to reverse the spell.
2013	Frozen	101	0	Fearless **Anna** sets out to find her sister **Elsa**, whose icy powers keep the kingdom of Arendelle in eternal winter. She is accompanied on her adventurous journey by Kristoff, a young iceman and rugged outdoorsman with his reindeer, Sven, and the jolly snowman Olaf.
2015	The Good Dinosaur	94	6	The young, shy Apatosaurus **Arlo** accidentally falls into a river and is swept away. On his way back to his family he is accompanied by **Spot**, a young human cave-dweller. Arlo learns to overcome his fears and realises what he is capable of.
2021	Raya and the Last Dragon	107	0	The brave warrior **Raja** embarks on an exciting and dangerous journey to track down the last remaining dragon and save Kumandra (a country comprised of five separate kingdoms).

The orphaned protagonist is set in bold. Films are listed by year of release (Source of the descriptions: Back cover of the purchased German DVD).

**Table 2.** Descriptive Overview of the Portrayal of death in the films.

Title of films	Death of figure and orphan (All deceased person had a minor character status)	Depiction of death^a^	Emotional reaction	Death causality
Bambi	Bambi’s mother is killed by a hunter.	Explicit (in sound)	Negative	Murder
The Fox and the Hound	The mother of Tod, a baby fox, is killed by a hunter.	Explicit (in sound)	Not Depicted	Murder
The Lion King	Simba’s father dies by murder disguised as an accident. The ambush was set up by Simba’s Uncle Scar.	Explicit	Negative	Murder
The Hunchback of Notre Dame	Quasimodo’s gypsy mother dies defending him as a baby from the court and the law.	Explicit	Not Depicted	Murder
Mulan	The father of juvenile minor character Captain Li Shang dies in the war.	Implicit	Negative	Murder
Tarzan	The parents of Tarzan (an infant) are killed by a leopard.	Implicit	Not Depicted	Murder
Atlantis: The Lost Empire	The mother of Kida disappears in a column of light and “dies” in the sense that she disappears forever. The parents of Milo also died in his childhood, but this is not shown in the film.	Implicit	Negative	Others
Finding Nemo	Nero’s mother of Nemo dies in a fish attack when he is still a fingerling.	Implicit	Not Depicted	Murder
Brother Bear	Koda’s mother is killed by Kenai in a fight. Kenai’s parents are also dead at the outset of the film. In addition, Sitka, Kenai’s brother, is killed in a fight with Koda’s mother.	Explicit	Negative	Murder
The Princess and the Frog	Tiana’s father dies when she is between a toddler and a teenager. The death is not shown, but results from the plot. The causes remain unnamed.	Implicit	Not Depicted	Unknown
Frozen	The parents of the two girls Elsa and Anna have died in a shipwreck.	Implicit	Not Depicted	Accidental
The Good Dinosaur	Arlo’s father dies in a storm while saving his son. The parents of Spot are also dead, but this is not shown in the film.	Explicit	Negative	Accidental
Raya and the Last Dragon	Raya’s father sacrifices himself to save her from the Druun (spirits that turn everything to stone) and is turned to stone. In the end, Raja and her friends manage to revive everyone through the reassembled dragon stone.	Sleep	Negative	Others
Total		Implicit = 6		Unknown = 1
		Explicit = 6		Accidental = 2
		Sleep = 1		Murder = 8
				Others = 2

Films listed by year of release.

a The death status was permanent/final except for ‘Raya and the Last Dragon’, where he was resurrected as the same person.

### Qualitative Analysis with the QUAGOL

We used the QUAGOL (Dierckx de Casterlé et al., 2012) to deepen our understanding of how death is portrayed in Disney and Pixar films. The QUAGOL is an operationalised analysis tool based on the Grounded Theory method (Corbin & Strauss, 2008), which promotes the recognition of the meaning of statements and facilitates a holistic and integrated understanding of the data. According to the QUAGOL, a step-by-step, repetitive approach with systematic coding encourages creativity and enables deeper insight into the data.

The research team consisted of six researchers with health science backgrounds, three of whom are also active in the field of biomedical ethics. Using the QUAGOL, the subject presented in one film was repeatedly compared to the other films until saturation was reached. All researchers were trained in the QUAGOL method by the lead researcher before beginning the analysis.

The analysis process was structured by factsheet forms designed by the lead researcher following the QUAGOL. The factsheet was used to guide the researchers in summarising the most important aspects and questions, and included the possibility of entering free text. In six project meetings and five independent work sessions, each of the six researchers was assigned to watch and analyse four films. The films were exchanged during the analysis to ensure that they were analysed independently by more than one person. Data was shared in team meetings conducted in person, and the data from the factsheet forms were subsequently consolidated. The films were all purchased in Switzerland. Although they could be played in different languages, the quotations used in the study were taken from the English subtitles.

Following QUAGOL, a formal peer debriefing was conducted and the findings reviewed by two individuals: the first an artist-lecturer at the Zurich University of the Arts with a specialisation in film studies, and the second an individual who lost both parents as a child, had watched many films as a child, and viewed the same all again for this study. Both reviewers’ written feedback was incorporated into the final text, which was once again approved by all team members before publication.

### Quality Control

To maintain oversight for the entire study the leading author reviewed all films. In addition, to ensure reliability, each film was independently viewed or coded by two separate members of the research team. As a result, each film was viewed, analysed and coded by at least three researchers, and its coding results discussed by the entire group.

### Ethical Considerations

Under Swiss law, this study required no formal ethical approval by a research ethics committee. All the children’s films are freely available and either released without age restriction or as suitable for children aged six and over.

## Results

### Quantitative Study

#### Film Selection

We included the 57 films identified in Tenzek and Nickels (2019), along with 18 additional films released since 2015. A total of 75 Disney and Pixar films were considered, of which 13 met the inclusion criteria, of which two were released after 2015. From 1937 to 2022, 17.3 % of all Disney films portrayed the death of one or both parents. Table 1 shows the characteristics and findings from the included films.

#### Film Characteristics

The parents whose deaths are depicted are all cast as minor characters (see Table 2). Two deaths are accidents that might have been avoided (Frozen and The Good Dinosaur), two parents are killed by a wild animal (Tarzan and Finding Nemo), three are shot by a hunter (Bambi, The Fox and the Hound and Brother Bear), and one falls in war (Mulan). In one film, the cause of death remains unknown (The Princess and the Frog). Two deaths seem to serve a higher purpose (Atlantis: The Lost Empire, and Raya and the Last Dragon). In The Lion King and The Hunchback of Notre Dame, the death of the parents by murder is explicitly depicted and the dead parent is represented by a motionless body. Only in one film (Raya and the Last Dragon), is death portrayed as a reversible state, whereas in all other films, death is depicted as a permanent, irreversible state. In all films, the surviving children react as expected, with negative emotions such as sadness, grief, or anger. In two films, a minor character is portrayed as happy about the parents’ death (Uncle Scar in The Lion King and Judge Claude Frollo in The Hunchback of Notre Dame).

### Qualitative Study

**Figure 1.**
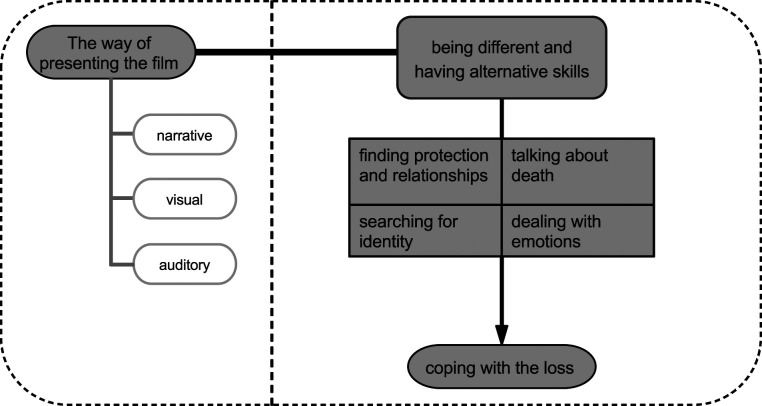
The seven concepts on the Effects and Reactions of the orphan and the environment (Source: Authors’ elaboration).

The qualitative analysis identified seven concepts relevant to the representation of death and the impact on the child protagonist, as identified in the diagram below (Figure 1). ‘The way of presenting the film’ narratively, visually and aurally has an effect on the experience of all plot elements. The child identifies through the experience of being different and having alternative skills and has the goal to of coping with the loss, which is evident throughout all the stories and it is also the overarching theme from which something can be learned in all the films. This learning occurs through the four concepts shown in the middle. The child protagonists seek to find protection and relationships and are searching for their identity. All the films show how to talk about death (when sensibly adapted to the situation) and an adequate approach to dealing with emotions can make a positive contribution to the ongoing process of coping with the loss and thus create the conditions for a brighter future. Figure 1 displays an overview of the concepts identified.

#### The Representational Techniques

The choice of images and music, in addition to and in support of content, plays a decisive and varying role in the effect on the viewer of the multi-layered depiction of death. Depending on the personality and biography of the viewer, the scenes and the film may have an effect specific to the individual. The images rendered differ greatly according to the year of the film. The newer the film, the more realistic the drawings and computer animations. Films have a high potential to transport impressions, emotions, and content, whereby music seemed to have a bigger emotional impact on viewers than images. Although many of the films communicate with spoken language, some use a combination of evocative images together with emotional music to convey meaning (e.g., Bambi). Symbolic events and emotions can be represented by a specific sound, indicating to the viewer what to expect in the next scene. The death scene is portrayed dramatically in six films. In The Lion King, for example, the father falls off a cliff while rescuing his son and is trampled to death by a herd of wildebeest as his son looks on.

In most films (see also Table 2) death is presented initially as a brief shock and as an ordinary part of life. Although the death of a parent has an impact on their subsequent circumstances and life course, many orphans continue their lives seeming surprisingly unharmed in the end. Although the deaths are portrayed as profoundly sad, in each film there is also always a character who lightens the mood with jokes and good spirits (see Table 3).

**Table 3.** Influence of death on the Orphan or the Plot of the film and Sidekicks.

Film	Friends and sidekicks
**The death of the parent is hardly addressed in the film, the half-orphans seem to cope well with it.**	
Bambi	Thumper and Flower, friends of Bambi
Mulan	Mushu and Cri-Kee as Mulan’s companions
Finding Nemo	Dory (a regal blue tang) accompanies Nemo’s father on his search
**The death of the parent is addressed in the film but has little influence on the plot itself and the half-orphans seem to cope well with it.**	
The Fox and the Hound	Dinky (a finch) and his friend Boomer (a woodpecker) and the caterpillar that they try to eat
Tarzan	Terk and Tantor, Tarzan’s best friend
Atlantis: The Lost Empire	Four supporting characters who accompany Milo
Brother Bear	Rutt and Tuke (Canadian moose) as supporting characters
The Princess and the Frog	Ray (Cajun Firefly) as the supporting character
**The death of the parent is a recurring theme in the film and influences both the plot and the half-orphans.**	
The Hunchback of Notre Dame	Victor, Hugo, and Laverne (three gargoyle statues) as friends of Quasimodo and Clopin Trouillefou (a puppeteer) as the storyteller
Frozen	Olaf, a snowman created by Elsa and also a friend of Anna, and Sven
Raya and the Last Dragon	Raya’s best friend Tuk Tuk (a mix of armadillo and pill bug), the dragon Sisu (a divine water dragon), and Little Noi (a toddler con artist)
**The death of the parent is a recurring theme in the film and influences the plot. The half-orphans are burdened by the consequences.**	
The Lion King	Timon and Pumbaa, friends of Simba. Rafiki the shaman, an old mandrill
The Good Dinosaur	Forrest Woodbush (a Styracosaurus*)* as the supporting character

Films listed by year of release.

##### Finding Protection and Relationships

Children portrayed as losing one or both parents are particularly vulnerable because of this experience. They need protection and seek that protection from a variety of sources. They often seem to be looking for someone to stand by them, their surviving parent, a friend, a peer, or another adult who is like a mentor. Basic trust in relationships does not always come easy for orphans. For example, Raya and the dragon Sisu in human form philosophise about how hard it is to be human and about unpleasant experiences. Raya states that the world is broken and that no one can be trusted. She talks about her disappointing experience in a “world of orphans” and about how sad the world is because of greed.

Sisu to Raya: “Or maybe the world’s broken because you don’t trust anyone. (54:16) … (55:73)…if you wanna get someone’s trust, you have to give a little trust first.” (Raya and the Last Dragon 54:16)

It is surprisingly rare for a surviving parent to be portrayed as a protector and supporter of their child. Surviving parents are portrayed as challenged by the care of their children for a variety of reasons, many of which are beyond their control. The difficulty of coping as a single parent is also succinctly portrayed in The Good Dinosaur: After the death of their father and before Arlo himself falls into the river and is swept away, Arlo’s mother needs her children’s help with the chores previously done by her husband to meet their basic needs:

Momma Ida, Arlo’s mother: «I know it’s hard without Poppa, but I need you to do more, Arlo.»

Arlo loads up more grain: «Don’t worry, Momma. I won’t let us starve.»

«You’re a good son.» (The Good Dinosaur 00:19:58)

Sometimes, the child turns to friends to get through this difficult time. In Tarzan, for example, in addition to his friends Terk and Tantor, his relationship with Jane is also portrayed in great detail. This helps him realise why he felt out of place. Tarzan and Jane build a solid relationship and learn to trust each other. Siblings, who appear in three films, were also shown as a source of support. An example of this is the film Frozen, which shows the relationship between the sisters Anna and Elsa. Although their relationship is distant at first, they eventually grow closer and begin to support and protect each other.

The Disney and Pixar films typically have happy endings. Except for the Hunchback of Notre Dame, the main characters find a new family through a romantic relationship, or the lost orphans find their family again. Table 4 shows the relationship between the main character and their bereaved parent, friends, and non-parental adults in the films studied.

**Table 4.** Relationship With the Surviving Parent, Friends, and Non-parental Adults.

Film	Family	Friends of more or less of the same age	Non-parental adults
	Surviving parent (mentioned Siblings)	1 = close relationship	1 = friendly and protective
		2 = also orphan	2 = malicious
			3 = mentor, not a caregiver
		3 = orphan falls in love with	0 = nothing special/explicit
Bambi	Barely present (0)	1) Thumper and Flower	0)
		3) Faline, a female deer	
The Fox and the Hound	Unclear (0)	1) Copper	1) Widow Tweed
		3) Vixey a vixen	3) Big Mama the owl
The Lion King	Does not know that the child is still alive (0)	1) Timon, Pumbaa	2) Scar
		3) Nala, a lioness	3) Rafiki
The Hunchback of Notre Dame	Not present (0)	1) Esmeralda	2) Judge Claude Frollo
Mulan	Unclear (0)	1) Mulan	0)
		3) Mulan	
Tarzan	Both deceased (0)	1) Terk and Tantor	1) Tarzan’s adoptive parents, the gorillas Kala and Kerchak
		3) Jane Porter	
Atlantis: The Lost Empire	Supportive (0)	2) Milo Thatch	0)
		3) Milo Thatch	
Finding Nemo	Overprotective (All deceased)	1) The Tank Gang: fishes living in an aquarium in a dental clinic	3) Gill, a scarred moorish idol fish
Brother Bear	Barely present (0)	2) Kenai	3) Tug, a wise old grizzly bear
The Princess and the Frog	Supportive (0)	1) Louis, an alligator and Ray	3) Mama Odie, a blind, old voodoo priestess
		3) Prince Naveen	
Frozen	Both deceased (Anna and Elsa)	1) Olaf, Kristoff and Sven	2) Hans
		3) Anna and Kristoff	
The Good Dinosaur	Does not know that the child is still alive (Two)	2) Spot	3) Butch, a Tyrannosaurus rex
Raya and the Last Dragon	Unclear	2) Little Noi and Boun	3) Sisu

Films listed by year of release.

##### Searching for Identity

In some films, the child has a predetermined role or responsibility to fulfil. For instance, Elsa, Simba, Kida, and Bambi have a duty to rule a kingdom. The children with responsibility are aware of these expectations before their parents’ deaths, but are free to grow into their roles, a process which includes rebellion, forming their individual identities, negotiating their responsibilities with their parents, and dealing with uncertainty. When the death of a parent occurs, the child is suddenly expected to fulfil these responsibilities. The children in the films respond in two ways: They can either overcompensate (e.g., Tiana and Li Shang, who both want to do the “assumed task” with excellence, without regard to their developmental needs) or refuse the role completely (e.g., Elsa and Simba, who run away from their families and kingdoms). Initially, children often find it difficult to fill their new role and feel both inadequately prepared and insecure about their abilities in absence of parental support. Over time, they gain experience and maturity. They are then able to balance their identity with the responsibilities of their new role and develop the necessary skills.

Poppa Henry, Arlo’s father: «It’s okay, Arlo, I’m sorry. I just wanted you to get through your fear. I know you have it in you.»

Arlo: «But I’m not like you.»

Poppa Henry: «You’re me and more» (The Good Dinosaur 00:18:13)

The children portrayed in the film develop the confidence to face more complex situations by exposing themselves to unfamiliar situations and dealing with them successfully. They learn to trust their own decisions, even where this means deviating from societal expectations. Learning to trust themselves and their decisions eventually enables them to take on the role expected of them. A good example is Simba, who recognises that he has the skills within himself and that by expressing and developing these skills, he is able to fill the role of King that he had once rejected out of insecurity.

The effect on the portrayed child is different if they did not know their parents well. These children do not orient themselves towards their birth parents; therefore, they do not see it as their task to fulfil their parents’ legacy. Instead, their sense of identity is rooted in their new environment with an adoptive family. They may work harder than others in their social environment to ‘earn’ acceptance and willingly accept poor treatment in return for that acceptance. Because they behave in a way that prioritises social acceptance, it sometimes seems as if they have to suppress parts of their identity.

##### Being Different and Having Alternative Skills

The orphans featured in the films often stand out from the rest of society as different or special. This otherness includes the absence of a parent, lacking or having varying abilities, having special skills or disabilities, and growing up among another species. Although the death of their parents is often not the cause of this otherness, these children lack the parental protection that other marginalised children might often have.

Adapting and learning new skills serves as a survival strategy but seems to be coloured more by species differences and special needs than by the loss of a parent. For these children, belonging to a group seems to be paramount. Many orphans show a strong desire to regain their footing. Feelings of wanting to belong are expressed clearly in The Hunchback of Notre Dame. At the beginning of the film, Quasimodo, who calls Laverne (a gargoyle) his friend, is on the tower of Notre Dame marvelling at the preparations for the Feast of Fools when the following exchange occurs:

Laverne: «Well, did ya ever think of goin’ there instead?»

Quasimodo: «Sure, but I’d never fit in out there. I’m not normal.»

Laverne: «Oh, Quasi, Quasi, Quasi.» (The Hunchback of Notre Dame 00:08:20)

In general, the desire to belong to a social group is fulfilled in many films when the protagonists regain the ability to love and forgive. This seems to be a central moment of healing in overcoming grief and frustration. The inner process of accepting oneself as one who is coping with emerging conflicts and developing the necessary skills is portrayed clearly in all the films. This character development constitutes the ‘happy ending’ in these Disney films.

##### Talking About Death

The communication between the characters regarding death is often non-verbal; this proves nevertheless effective. At the same time difficult but important things are often not addressed, which creates uncertainty and leaves room for frightening interpretations that can be more troubling than reality. Not wanting or being able to talk about what happened can be observed among different characters.

Pumbaa: «What’d you do, kid?»

Simba: «Something terrible. But I don’t want to talk about it.»

Timon: «We don’t want to hear about it» (The Lion King 00:43:31)

In most films, the death of the parent is explicitly communicated or the orphan himself was present when the parents’ death occurred. In The Princess and the Frog, the father is simply not shown as the film progresses. In Frozen, the death of the parent is confirmed non-verbally when the ship is shown to sink during a storm and the family portrait is simply covered with a curtain. In Brother Bear, the orphan only learns at the end of the film that his mother has died.

The decision to withhold an open, honest, clear, and comprehensive communication with a child at times results in adverse consequences, particularly in relation to the child’s understanding of and coping with the parent’s death. The missing information is sometimes replaced by their imaginary version of events. A lack of understanding of the death of a parent can lead the child to feel insecure and misinterpret the events. A child’s ability to verbalise their perception of a parents’ death seems to be a sign of maturity and indicates that the child has processed and accepted the parents’ passing. Table 5 shows the communication of and reaction to the parental death.

**Table 5.** Communication and Reaction to the death.

Film	Communication of the death to the audience	Communication of the death to the orphan	Reaction of the orphan
Bambi	Bambi and his mother are on the run from hunters. A gunshot sounds, and from that moment on the mother is no longer seen.	His father tells him	His eyes show sadness, he seems incredulous and lowers his head, has a tear in his eye, walks away with his father.
The Fox and the Hound	The mother is seen fleeing with Tod in her mouth, dogs are barking. After hiding him by a fence, she lovingly says goodbye, we see her disappear behind a hill and hear two gunshots.	It remains unclear throughout the film whether he understands that his mother has died.	Fearful and shy as the owl Big Mama finds him and he snuggles up to her.
The Lion King	Simba sees his father fall from a rock. Simba does not realise that his uncle has caused his father’s death. He lies motionless on the ground.	When Scar speaks up, Simba seems emotionally overwhelmed, with wide eyes and tears.	He realises that his father is not responding, tries to wake him up, and then snuggles up to him, crying.
The Hunchback of Notre Dame	The mother is pushed by Frollo, she falls and remains motionless.	He is an infant when the parent dies and grows up with the fact.	He does not perceive the death.
Mulan	A battlefield is seen. A soldier approaches with the father’s helmet.	Seems to understand it immediately.	Seems thoughtful, moves away from the crowd, puts his sword in the snow and the father’s helmet on it, and bows. Shortly afterwards, he continues his mission and seems stronger.
Tarzan	The Leopard is seen chasing the gorilla cub, then falls silent. The next day, the gorilla mother hears a baby crying and enters the devastated tree house. The rifle is on the floor and the motionless bodies of the parents can be seen behind a board. They appear blurred, without heads or hands. Bloody paw prints are also visible.	He is an infant when his parents die and grows up with the fact.	He does not perceive the death.
Atlantis: The Lost Empire	The mother disappears upwards into a column of light.	She is still a child and grows up with the fact.	She cries, calls for her mother, stretches out her hands, and falls to her knees.
Finding Nemo	A Baraccuda arrives, a short fight ensues and father Marlin is knocked out. When he regains consciousness, the only thing left is Nemo as a spawning ball with a flaw on it.	He is an spawning ball when the parent dies and grows up with the fact.	He does not perceive the death.
Brother Bear	During the fight, the mother bear lunges at the spear. You only see the rock and hear her, then you see Kenaj crawling out from under the lifeless body.	Kenaj confesses to Koda that Koda’s mother died fighting him and that she will never come back.	Koda’s eyes open wide. With tears in his eyes, he says “No”, first questioningly, then firmly and painfully, and runs away. He flees to a tree and cries briefly.
The Princess and the Frog	In the opening sequence of the film, the father can be seen with his family. The film begins with a time leap. Tjana has grown from a girl into a young woman and her father is no longer present. Later in the film it is mentioned that he has died.	It is not shown.	It is not shown.
Frozen	The parents’ ship is seen sinking in the waves of the storm. The image of the parents is obscured by a black curtain and the funeral is shown.	It is not shown.	Anna is seen at the funeral, then outside Elsa’s door, singing and cowering on the floor. Elsa sobs and crouches on the other side of the door.
The Good Dinosaur	When the tidal wave comes, the father saves Arlo by throwing him onto a higher rock. He slips off and disappears into the waves. The screen is black, then you see the gravestone and Arlo’s mother says that she knows it’s hard without his father.	Seems to understand this immediately.	He fearfully cries out for his daddy before the latter is swept away by the waves. In the next scene, he helps his mother to collect the harvest.
Raya and the Last Dragon	The father is seen to turn to stone after giving Raya the task of saving the people and bidding her a loving farewell.	Knows it the moment when the father turns to stone.	She falls into the river with her eyes wide open and seems distraught. As she calls for her petrified father, she seems sad, gasps, and whimpers.

Films listed by year of release.

##### Dealing with Emotions

Powerful emotions are framed in the films by emotive music and accompanied by touching images. The initial reactions of the orphans are described in Table 5. How the emotions are shown depends on the theme of the film and the circumstances in which the parents died. When a parent dies, films usually show how the orphaned child processes grief, guilt, loneliness, and lack of parental support. The child often expresses these feelings by being afraid, withdrawing from friends and family, and crying. All the orphans in the film eventually learn to cope with loss and loneliness. They are often guided by a caregiver, who is often a person outside the immediate family. This ties into the larger theme in most films about children growing up and finding their own way through life. Death is portrayed as a natural part of life, with periods of mourning and missing loved ones. Grief is portrayed as a brief moment of crying, fear, or loneliness.

In the wake of grief, many children are shown to struggle with trusting others and being vulnerable. In two films, feelings of guilt especially stand out. In The Lion King and The Good Dinosaur, the lead characters repress their feelings, feel ashamed and angry, and blame themselves for the death of their parents. For example, Simba is convinced that it is better to live a completely different life, away from the memories of his childhood and his family. Although there are subtle differences between all the films in this regard, they generally show the emotional challenges and triumphs as the child matures and learns to handle life’s challenges gracefully.

##### Coping with the Loss

In the majority of the films, coping with parental loss is shown to be adequate and even exemplary, as observed by Corr (2004), Graham et al. (2018) and Arruda-Colli et al. (2017). As described above, many protagonists return to a normal and satisfying life relatively quickly, often as soon as they have completed a pre-determined “task” that must be fulfilled. The initial coping strategies shown in Table 2, whether isolation, displacement, or running away, never prove to be fully effective. An effective coping strategy requires the child to confront their grief and subsequently accept their situation. Doing so allows the character to mourn and finally move on and mature. Only in two films, Frozen and The Lion King, is the pattern of mourning longer and correspondingly more complex. For example, in The Lion King. Simba suffers deep feelings of guilt, as he wrongly thinks he is responsible for his father’s death, complicating the grieving process.

Maladaptation and parentification, i.e., the assumption of the parents’ tasks, can be seen in many of the films, however, the process of developing a sense of trust and belonging eventually allows the child better to cope with their parents’ death. For example, in trusting his relationship with Nala, Simba releases the heavy burden of his difficult past and feels a sense of protection that allows him to be himself again.

## Discussion

This study has identified Disney and Pixar films that show the death of one or both parents and analysed how situations of loss and the ensuing experiences are described and portrayed. Parental absence as a result of death is a common dramatic theme in children’s films, providing child protagonists with significant problems to overcome and providing the foundation for adventure to develop within the storyline (Colman et al., 2014).

Similarly to other studies (Bridgewater et al., 2021; Colman et al., 2014; Graham et al., 2018; Lammon, 2019, 2022; Sedney, 2002), our findings indicate that films that depict appropriate grief responses may help children to gain a deeper understanding of the meaning of death and to cope better with the challenges of parental death. For example, many films show how the orphaned child experiences the natural emotions of grief, guilt, and loneliness. The child expresses these feelings by being afraid, withdrawing from friends and family, and crying as a natural part of the grief response. With time and experience, they then learn to cope with their loss and loneliness. Watching these films may help children better understand and adjust to death. At the same time, the depictions of death in films could have detrimental effects on children (Lammon, 2023). They may be prompted to worry about the reoccurrence of similar events within their own family. To counter this, Colman et al. (2014) recommend watching these films with an adult who can provide emotional support to children during the inevitable horror of murder and mayhem scenes.

The cause of death may be significant to children, who may recognise a similarity to the death of their own parents. In the films included here, the parental death is most commonly the result of murder or an accident, a fact that bears no relation to statistical reality. In Switzerland, for example, of the 71,192 parental deaths in 2021, only 5.5% were due to accidents and violence (Office of Federal Statistics, 2023b; 2023c), and only 18 non-suicidal deaths resulted from firearms (Office of Federal Statistics, 2023a). Colman et al. (2014) compared 45 top-grossing animated films for children with 90 top-grossing dramatic films for adults and found that death and murder were shown more frequently in children’s films. Nonetheless, the cause of death plays a questionable or smaller role in the grieving process than might be assumed (Alvis et al., 2023; Kaplow et al., 2014; Stroebe et al., 2007; Weiss, 2006).

Our findings show that in the films there is often no parent at the child’s side, whether one survives or not. The children thus seek an alternative caregiver, even though basic trust in relationships does not come easy to them. Where a surviving parent becomes the most important caregiver, the better the bond with the surviving parent, the better the inner image and sense of connection with the deceased parent. Acceptance of death seems more possible when a reliable caregiver is available (Alvis et al., 2023; Ryan & Ripley, 2023; Weiss, 2006). At the same time, it can be a relief to parents to know that children can seek out alternative caregivers to the surviving parent, and that they can be supported in finding such a person who is not unduly burdened by the tragedy. A network providing the appropriate caregiving support in case of need is important in supporting grieving families (Koblenz, 2016).

Belief in untrue assumptions about the death can hinder the grieving process by promoting feelings of guilt and preventing acceptance of the loss (Alvis et al., 2023; Weiss, 2006). The fulfilment of dreams shown in films can be helpful and provide temporary comfort if children have a loving and consistent caregiver at their side to help them cope with their thoughts and feelings (Alvis et al., 2023; Ellis et al., 2013; Graham et al., 2018; Greeff & Human, 2004; Karydi, 2018; Luecken & Roubinov, 2012; Sedney, 2002). Grief can be dealt with in a multitude of ways, and strategies to cope with grief are best when chosen to suit the individual. The early loss of a parent can trigger a process allowing children to overcome later adversity, adapt to changes, and give life meaning (Koblenz, 2016).

Attempting to be ‘normal’ is often used as a coping strategy by the children in the films. All orphans realise that they are different and are treated differently, so that they try to restore normality by dealing with their otherness and integrating it into their own story. This otherness includes the absence of a parent, lacking or having varying abilities, or growing up among another species. In accordance with our results, Metzing-Blau and Schnepp (2008) report that children living with seriously ill parents make an effort to keep the family together and to continue living as normally as possible. This effect can encourage bereaved children who watch the film to reflect on their own desire to be normal, or it can be used by adults to start a conversation about the topic of ‘being different', which is often portrayed as something positive at the end of the film.

Grief and loss are not only psychological processes but manifest themselves through communication, especially within the family itself (Barboza & Seedall, 2021; Bosticco & Thompson, 2005). How death is communicated varies across the films. Films that depict death can stimulate conversations about it (Cox et al., 2005). Fearnley (2010) states that even professional avoidance of speaking openly to children or using euphemisms may be an indication of personal difficulties and the general attitude of our society and culture towards death. It is easier and often more comfortable not to name grief and thus to ignore “the elephant in the room”. Professionals avoid talking about death with those affected, especially children, in order not to burden or worry them, and as a result often do not recognise children’s need for information. While the needs of children who are experiencing or have experienced the death of a parent are often overlooked and not addressed with the urgency and directness they deserve, children who are regularly informed and involved in conversations about their current situation during the dying phase and after the death of a parent are better able to deal with the uncertainty and upheaval in their lives (Alvis et al., 2023; Angelhoff et al., 2021; Fearnley, 2010; Ratcliffe & Byrne, 2022; Zajac & Boyatzis, 2021). Our findings indicate that these films can afford parents the opportunity to initiate an educational conversation about death. Colman et al. (2014)likewise indicate that films can give children significant insights into loss-related grief and a deeper understanding of dying.

Films offer great potential to overcome the taboos surrounding death. This knowledge of the impact of films implies a certain responsibility on the part of the film industry representing the topic for their target audience. It is likewise clear that many children and young people watch films, and family members and professionals should accompany them in a meaningful way. The importance of the effect of fiction on coping and behaviour becomes particularly clear when one considers that the main characters in children’s films have a higher risk of dying than in dramatic films for adults (Colman et al., 2014). Bohrmann (2018) describes entertainment through films as a multi-layered phenomenon in which the viewer is actively involved. Films are more than mere entertainment; as ethical narratives, they pose questions about proper moral behaviour and encourage the viewer to reflect and empathise. The intense immersion in the film plot can blur the difference between fiction and reality (Bohrmann, 2018). Vaage (2007) and Jaén Portillo (2024) emphasises that viewers can develop an imaginary closeness and emotional sympathy with the film characters. This empathy makes it possible to empathise with the characters and understand their actions. One’s own memories and the consternation triggered by empathy can lead to self-reflection. The imaginative experience of watching a film can encourage viewers to examine their own goals and desires and think about the relevance of events to themselves. Through this self-reflection, the viewer leaves the fictional world (Vaage, 2007).

### Limitations and Further Research

Firstly, this study focuses only on Disney and Pixar films, so that the results cannot be generalised as applying to all animated films. Secondly, only films in which parental death occurs during the film were included. Subsequent studies could look at films that have death itself as a major theme, such as “Lilo & Stitch” and “Big Hero 6”, or where a child is known to be an orphan but the death does not occur during the running time of the film. Thirdly, although the analysis based on QUAGOL provides deep and broad insight, the data were collected with the involvement of experts. The educational benefits and psychological impact on the target population itself (children having suffered parental loss) were not studied in an applied way. Although direct observation of viewers of different ages and biographies, both while watching the film and in the subsequent discussion, would be preferable, such an intervention shows itself to be particularly sensitive. It reveals the vulnerability of the target population, which must be explicitly considered in study design and corresponding precautionary measures taken (in terms of psychological support, and parental or peer involvement). We hope our findings will encourage further similar research to explore the unmet needs of children after parental loss.

## Conclusion

This study identifies and describes how films portray the death of parents, the children’s reactions to the loss at the moment of death, and the impact of death on children’s future lives. In addition, aspects of resilience and bonding with other people have been explored. Communication is shown to play a pivotal role in integrating this difficult situation into one’s biography. These films can provide an opportunity for affected families and professional caregivers to address and reflect on their own experiences and to speak with their children about the unspeakable. Further research is recommended to investigate the experiences of children and parents in watching these films.

## Appendix

### List of Films

Disney and Pixar films showing an end-of-life scene as reported by Tenzek and Nickels (2019) and films newly released between 2015 and 31.12.2022 (bolded), classified and arranged alphabetically.

**Table:**

***Included films in which the death scene of a parent is shown or occurs in the film***			
1	Atlantis: The Lost Empire	8	**Raya and the Last Dragon**
2	Bambi	9	Tarzan
3	Brother Bear	10	The Fox and Hound
4	Finding Nemo	11	**The Good Dinosaur**
5	Frozen	12	The Lion King
6	Hunchback of Notre Dame	13	The Princess and the Frog
7	Mulan		
***Excluded films - Being orphaned is only mentioned***			
14	Aladdin	22	Ralph Breaks the Internet
15	Cinderella	23	Snow White and the Seven Dwarfs
16	Dinosaur	24	Tangled
17	Frozen II	25	The Great Mouse Detective
18	Lilo and Stitch	26	The Jungle Book
19	Oliver and Company	27	The Rescuers
20	Peter Pan	28	The Rescuers Down Under
21	Pocahontas	29	The Sword in the Stone
***Excluded films - not an issue***			
30	101 Dalmatians	53	**Onward**
31	A Bugs Life	54	Pinocchio
32	Alice in Wonderland	55	Ratatouille
33	Beauty and the Beast	56	Robin Hood
34	Big Hero Six	57	Sleeping Beauty
35	Bolt	58	**Soul**
36	Brave	59	**Strange World**
37	Cars	60	The Adventures of Ichabod and Mr. Toad
38	Cars 2	61	The Aristocats
39	**Cars 3**	62	The Black Cauldron
40	**Coco**	63	The Emperor’s New Groove
41	Dumbo	64	the Incredibles
42	**Encanto**	65	The Little Mermaid
43	**Finding Dory**	66	Toy Story
44	Hercules	67	Toy Story 2
45	Home on the Range	68	Toy Story 3
46	**Incredibles 2**	69	**Toy Story 4**
47	Inside Out	70	Treasure Planet
48	Lady and the Tramp	71	**Turning Red**
49	**Lightyear**	72	UP
50	**Luca**	73	Wall-e
51	**Moana**	74	Wreck it Ralph
52	Monsters, Inc.	75	**Zootopia**
